# Calcifying aponeurotic fibroma of the finger in an elderly patient: CT and MRI findings with pathologic correlation

**DOI:** 10.3892/etm.2014.1838

**Published:** 2014-07-11

**Authors:** JUN NISHIO, HIDEAKI INAMITSU, HIROSHI IWASAKI, HIROYUKI HAYASHI, MASATOSHI NAITO

**Affiliations:** 1 Department of Orthopaedic Surgery, Faculty of Medicine, Fukuoka University, Fukuoka 814-0180, Japan; 2 Department of Pathology, Faculty of Medicine, Fukuoka University, Fukuoka 814-0180, Japan

**Keywords:** calcifying aponeurotic fibroma, CT, elderly, MRI, pathology

## Abstract

Calcifying aponeurotic fibroma (CAF) is a rare, locally aggressive fibroblastic lesion that occurs predominantly in the distal extremities of children and adolescents. In the present study, a case of pathologically proven CAF arising in the right little finger of a 69-year-old woman is presented. Physical examination revealed a firm, immobile, non-tender mass. Plain radiographs showed a faintly calcified soft tissue mass without bone involvement and computed tomography scans clearly demonstrated the presence of the lesion. Magnetic resonance imaging revealed that the lesion exhibited low to intermediate signal intensity on T1-weighted images and heterogeneous high signal intensity with small foci of low signal intensity on T2-weighted spectral presaturation with inversion recovery images. Contrast-enhanced fat-suppressed T1-weighted images demonstrated intense heterogeneous enhancement throughout the mass. The patient underwent an excisional biopsy. Histologically, the tumor showed a biphasic pattern, composed of a moderately cellular fibromatosis-like component and irregular calcified areas with polygonal epithelioid cells. There has been no evidence of local recurrence four months following surgery. To the best of our knowledge, this case report describes the oldest patient with this condition.

## Introduction

Calcifying aponeurotic fibroma (CAF), also known as juvenile aponeurotic fibroma, is a rare, benign fibrous tumor that occurs in the distal extremities, in particular on the palmar surface of the hand. It usually presents as a slowly growing, painless, firm mass in children and adolescents, with a slight male predominance (1). Due to its infiltrative nature, CAF has a high rate of local recurrence following surgical excision. Malignant transformation is extremely rare (2). In the present study, a case of CAF arising in the little finger of an elderly patient is presented, as well as a review of the literature.

## Case report

A 69-year-old woman presented with a 7-year history of a slow-growing, painless mass in the radial side of the right little finger at the proximal interphalangeal joint. There was no history of antecedent trauma. Physical examination revealed a firm, immobile, non-tender mass. Neurovascular examinations were normal and the results from laboratory tests were within the normal limits, including C-reactive protein levels and white blood cell counts. The patient’s past medical history was unremarkable.

**Figure 1:**
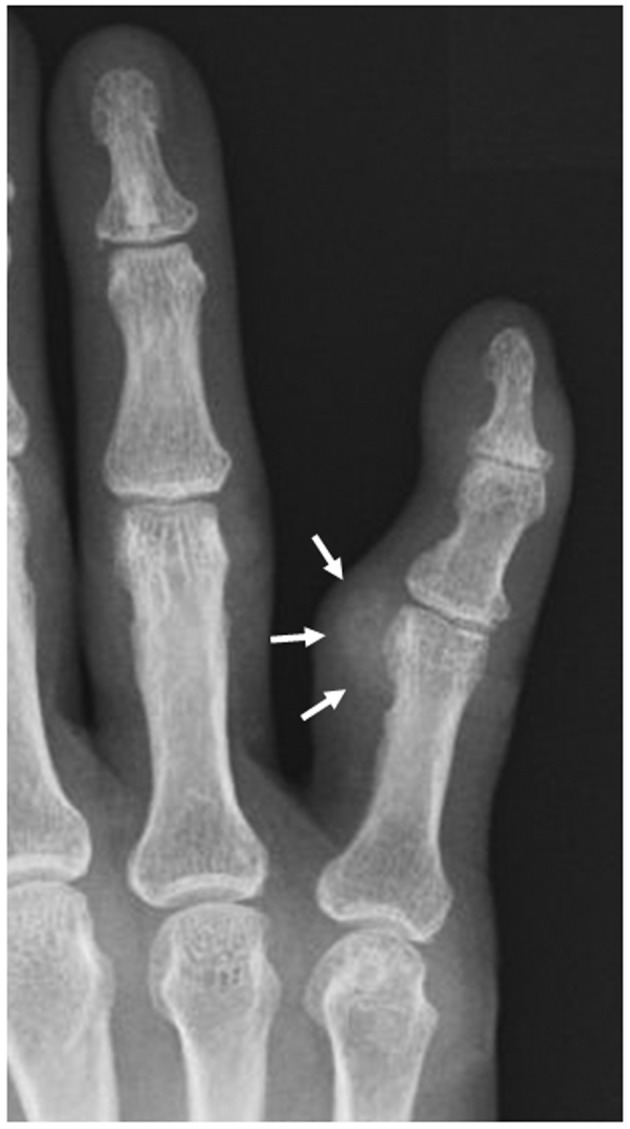
Plain radiograph reveals a faintly calcified soft tissue mass (indicated by the white arrows) without bone involvement.

**Figure 2:**
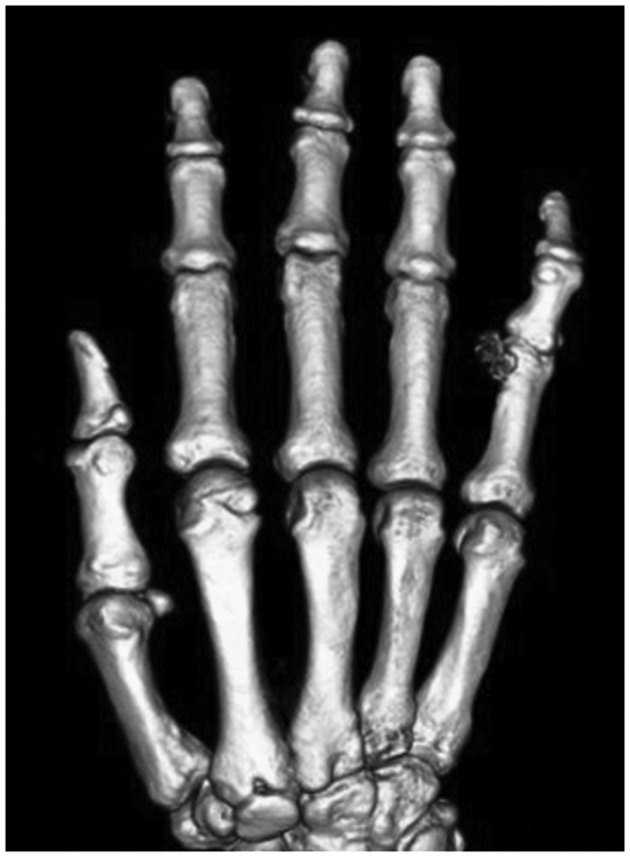
Three-dimensional computed tomography image demonstrating the presence of the lesion.

**Figure 3:**
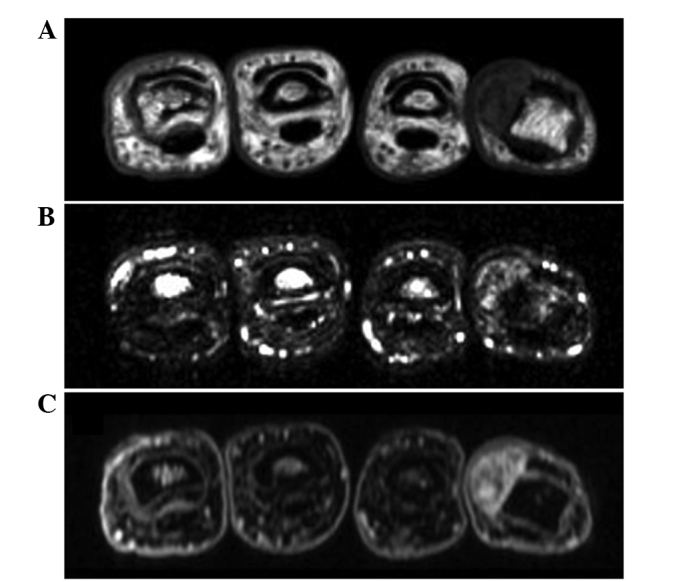
Axial magnetic resonance imaging showing a relatively well-defined soft tissue mass with (A) low to intermediate signal intensity on T1-weighted image and (B) heterogeneous high signal intensity with small foci of low signal intensity on T2-weighted spectral presaturation with inversion recovery image. (C) Contrast-enhanced fat-suppressed T1-weighted image demonstrates intense heterogenous enhancement of the mass.

Plain radiographs revealed a faintly calcified soft tissue mass without bone involvement (Fig. 1), and computed tomography (CT) scans confirmed the presence of the lesion (Fig. 2). Magnetic resonance imaging (MRI) exhibited a relatively well-defined soft tissue mass, 1.2×1.0×0.6 cm in size. The mass showed low to intermediate signal intensity on T1-weighted images (Fig. 3A) and heterogeneous high signal intensity with small foci of low signal intensity on T2-weighted spectral presaturation with inversion recovery images (Fig. 3B). Contrast-enhanced fat-suppressed T1-weighted images demonstrated intense heterogeneous enhancement throughout the mass (Fig. 3C). Based on these findings, the patient was diagnosed with a benign soft tissue tumor, including soft tissue chondroma.

**Figure 4:**
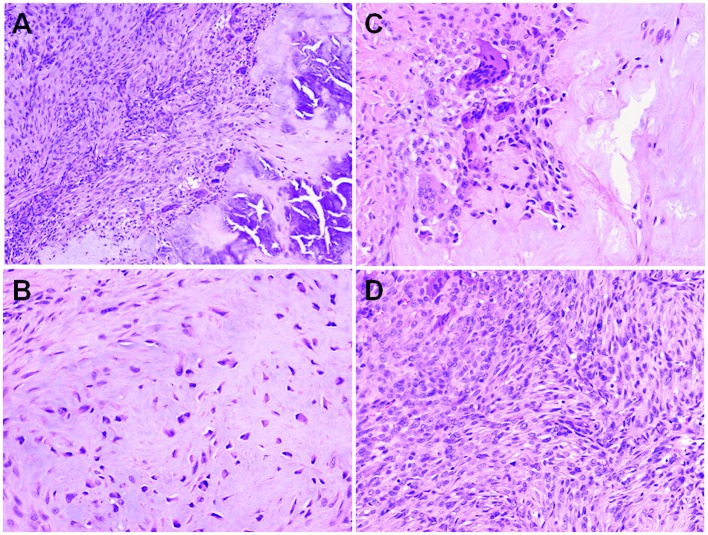
Histopathologic findings of calcifying aponeurotic fibroma. (A) The tumor shows a biphasic pattern composed of a spindled fibroblastic component and nodules of calcification. (B) Cartilaginous metaplasia is observed in the calcified areas. (C) Multinucleated osteoclast-type giant cells are seen around the calcified areas. (D) The spindled fibroblastic component is moderately cellular; however, atypical mitotic figures are not observed.

An excisional biopsy was performed under general anesthesia with tourniquet control. A midlateral incision was made on the radial side of the right little finger. The mass was adherent to the surrounding fibrous tissues; however, the mass was fully excised. No adjacent bone involvement was noted. Histologically, the tumor showed a biphasic pattern, composed of a moderately cellular fibromatosis-like component and irregular calcified areas with polygonal epithelioid cells (Fig. 4A) and foci of cartilaginous metaplasia were observed in the calcified areas (Fig. 4B). Multinucleated osteoclast-type giant cells were found around the calcified areas (Fig. 4C). The proliferating cells did not exhibit cellular atypia or evident mitotic figures (Fig. 4D). The results from the histopathological analysis were consistent with CAF.

The postoperative course was uneventful, and there was no evidence of local recurrence four months following surgery.

Written informed consent for publication was obtained from the patient and this study was approved by the Institutional Review Board (Fukuoka, Japan).

## Discussion

CAF occurs in patients over a wide age range; however, it is most common in children and adolescents (1). The median ages for male and female patients are 11 and 12 years, respectively (3). To the best of our knowledge, the present case is the oldest reported case of CAF.

CAF grows in a diffuse, poorly circumscribed manner and is often attached to the aponeurosis, tendons or fascia. Complete local excision is the treatment of choice. The histological examination of the lesion reveals two components: i) fibromatosis-like spindle-shaped cell elements, and ii) nodules of calcification, accompanied by more rounded, epithelioid cells (1). Two phases have been described in the development of CAF (4); in the initial phase the tumor has an infiltrative growth and often lacks calcification, whilst in the later phase the tumor is more compact and nodular and exhibits a more prominent degree of calcification and cartilage formation, as seen in the present case.

The pathogenesis of CAF remains uncertain; however, a fibroblastic/myofibroblastic origin has been suggested (3). It has been previously demonstrated using immunohistochemistry that the tumor cells usually express vimentin and smooth muscle actin, but are negative for desmin (1), and these results are in accordance with this proposal.

Only a few studies have described the imaging features of CAF. Plain radiographs may show a nonspecific soft tissue mass, with a variable extent of finely stippled calcifications (5), and bone involvement is rarely observed. CT scans are useful in order to determine the calcified areas of the lesion and its association with the adjacent bone. On MRI, CAF typically appears as an ill-defined subcutaneous mass with intermediate to low signal intensity on T1-weighted sequences and heterogeneous high signal intensity on T2-weighted sequences (6). Prominent areas of globular low signal intensity may be seen on all MR pulse sequences, corresponding with the presence of calcification. CAF usually demonstrates intense heterogeneous enhancement following intravenous gadolinium administration (6). The imaging results from the present case study were consistent with the aforementioned findings.

In older patients, the distinction of CAF from soft tissue chondroma may be difficult. As with CAF, soft tissue chondroma occurs most commonly in the finger or hand, with no connection to the underlying bone (7). However, unlike CAF, soft tissue chondroma is typically well-circumscribed rather than having an infiltrative border. In addition, soft tissue chondroma is less likely than CAF to recur. On MRI, soft tissue chondroma usually appears as a well-defined mass with intermediate signal intensity on T1-weighted sequences and high signal intensity on T2-weighted sequences (8). The most important histological finding distinguishing soft tissue chondroma from CAF is the presence of infiltrating fascicles of fibroblasts at the periphery of CAF (9).

In conclusion, in the present case report the imaging findings of CAF with pathologic correlation in an elderly patient are described. Although rare, CAF should be considered in the differential diagnosis of elderly patients with a calcified soft tissue mass, in particular in the finger or hand.
